# Improvement of caproic acid production in a *Clostridium kluyveri* H068 and *Methanogen* 166 co-culture fermentation system

**DOI:** 10.1186/s13568-018-0705-1

**Published:** 2018-10-25

**Authors:** Shoubao Yan, Dong Dong

**Affiliations:** 1 School of Life Science, Huainan Normal University, Huainan, 232001 Anhui People’s Republic of China; 2 Anhui Yingjia Group Co., Ltd., Luan, 237271 Anhui People’s Republic of China; 3 Hefei Institutes of Physical Science, Chinese Academy of Sciences, Hefei, 230031 China

**Keywords:** Caproic acid production, Methanogenic bacteria, Co-culture fermentation, Scaling-up

## Abstract

The aim of this study was to develop a bioprocess capable of producing caproic acid using a binary fermentation system consisting of *Clostridium kluyveri* H068 and *Methanogen* 166 which could then be applied to the brewing of Chinese strong flavor liquor. We initially explored the mechanism by which the *Methanogen* 166 strain facilitates caproic acid accumulation, revealing its ability to convert accumulated H_2_ that is produced by *C. kluyveri* H068 into methane, thereby eliminating the hydrogen-mediated feedback inhibition that normally constrains *C. kluyveri* H068 and thus enhancing caproic acid production. In addition, laboratory experiments were conducted to optimize this binary fermentation system, allowing us to determine that the optimum conditions for caproic acid production are a mixed inoculum size of 10% with a *C. kluyveri* H588/Methanogen 166 inoculation ratio of 2:1 (v/v), a sodium acetate concentration of 20 g/L, a 4% ethanol content (v/v), and a yeast extract concentration of 10 g/L. We further scaled this optimized condition up to use in a 1000 L fermenter and the obtained caproic acid broth was subjected to pit-entry fermentation. Our results demonstrated that this pit-entry fermentation approach was an efficient means of improving the quality of Chinese strong flavor liquor.

**Electronic supplementary material:**

The online version of this article (10.1186/s13568-018-0705-1) contains supplementary material, which is available to authorized users.

## Introduction

Chinese liquor is a traditional alcoholic drink that is consumed widely in China and plays an important role in Chinese life and culture. Generally, it is typically classified into five categories based on its aroma: strong flavor liquor, light flavor liquor, sauce flavor liquor, sweet honey flavor liquor, and mixed flavor liquor (Li et al. 2013). Among these, strong flavor liquor is the most popular distilled liquor in China and it is characterized based on its aroma, mellow taste, and harmonious flavour. Chinese strong flavor liquor is traditionally fermented in a solid state system, in which pit mud serves as the base material for the resulting flavor. This mud is inhabited by various microorganisms, including bacteria that produce caproic acid, lactic acid, butyric acid, acetic acid, and methane. These microbes play vital roles in the production of aromatic compounds, and consequently give Chinese strong flavor liquor special characteristics that have been described by some as making it highly flavoured, sweet, and refreshing (Yan et al. 2015a). Of these microorganisms, those bacteria which produce caproic acid are among the most important for the overall fermentation process, as caproic acid reacts with ethanol leading to the formation of ethyl caproate, which is the major aromatic component of Chinese strong flavor liquor. Therefore, there is great industrial interest in improving the quality of Chinese strong flavor liquor via increasing the production of ethyl caproate. Typically a high caproic acid content will promote increased ethyl caproate production, conversely, a low concentration of ethyl caproate was produced. Caproic acid producing bacteria can also generate butyric acid, valeric acid, and other trace elements, and as such their metabolism can greatly influence the quality of the final liquor.

Methanogens are also an important group of microorganisms involved in the fermentation process, as they significantly contribute to the elimination of the “hydrogen inhibition” that will otherwise arise during traditional solid-state fermentation of Chinese strong flavor liquor (Wu 2002). Indeed, in traditional pit-based brewing environments, caproic acid has been reported to primarily form via mutual interactions between caproic acid-producing bacteria with methanogenic bacteria (Wu 2002). Although caproic acid bacterial can live alone as a mono-culture, they grow more rapidly and better produce caproic acid when co-cultured with methanogenic bacteria. It is therefore critical that such a co-culture system be investigated and optimized.

In present study, we developed a process for producing caproic acid via the co-culture of *C. kluyveri* H588 and Methanogen 166. We further conducted a preliminary investigation of the mechanism by which these methanogenic bacteria facilitate caproic acid accumulation. Fermentative assays were carried out to evaluate how inoculum size, strain ratios, initial concentrations of sodium acetate and ethanol, and addition of yeast extract affected resultant caproic acid production in this binary fermentation system. This allowed us to scale up the optimized fermentation conditions for use in a 1000 L pilot-scale fermenter. We additionally subjected the obtained caproic acid broth to traditional pit-entry fermentation in order to efficiently improve the quality of Chinese strong flavor liquor. This study helps to reveal the microbial interactions underlying the fermentation of Chinese strong flavor liquor and provides a promising means of enhancing caproic acid production that will be of value to the liquor brewing industry.

## Materials and methods

### Microorganism and culture maintenance

The *C. kluyveri* H588 strain (CICC 20008) used in this work was obtained from the China Center of Industrial Culture Collection (CICC, Beijing, China). The Methanogen 166 strain (ACCC 00138, a type of Methanobacterium) used in this work was obtained from the Agricultural Culture Collection of China (ACCC, Beijing, China). *C. kluyveri* H588 was maintained on modified Barker medium (MB; 1 L of the MB medium contained 5 g of CH_3_COONa, 0.4 g of K_2_HPO_4_, 0.2 g of MgSO_4_·7H_2_O, 1 g of (NH_4_)_2_SO_4_, 10 g of yeast extract and 20 mL of CH_3_CH_2_OH), sealed with paraffin oil, and kept at 4 °C. The *C. kluyveri* H588 inoculum was prepared using a seed medium containing the same components as MB except for 20 g/L yeast extract, and was grown in 500 mL serum bottles containing 450 mL of culture medium. Pre-cultivation was performed anaerobically at 35 °C for 7 days, and this culture broth was used as an inoculum for caproic acid fermentation experiments.

Methanogen 166 was anaerobically maintained and cultivated in MC medium. This medium contained (1 L) 0.5 g of K_2_HPO_4_, 7.5 g of NH_4_Cl, 0.2 g of MgCl_2_, 1 g of yeast, 10 g of CH_3_COONa, and 10 mL of CH_3_CH_2_OH (Jiang et al. 2002). After autoclaving, 0.6 g Na_2_S and 1.5 g Na_2_CO_3_ were added just prior to inoculations. The gas phase of the culture consisted of a mixture of hydrogen (80%, v/v) and carbon dioxide (20%, v/v). All pre-cultures were incubated anaerobically in serum bottles at 35 °C for 7 days, at which point the log phase of growth was achieved. All anaerobic procedures were carried out in an anaerobic chamber at a temperature of 25 °C under aseptic conditions.

### Caproic acid batch fermentation experiments

*Clostridium kluyveri* H588 mono-culture fermentation and co-culture fermentation in combination with Methanogen 166 were conducted anaerobically, with nitrogen gas used to fill void space in culture bottles (Ernest et al. 1985) and with an oxygen-free MB medium. These fermentation experiments were carried out under stationary conditions at 35 °C in 500 mL serum bottles each containing a 450 mL working volume. The initial inoculum concentration of *C. kluyveri* H588 and Methanogen 166 in all fermentation systems was 5% (v/v) and 5% (v/v), respectively, if not otherwise indicated. Samples were taken at regular intervals for the analysis of cell numbers and concentrations of caproic acid, butyric acid, H_2_, and CH_4_.

In addition, different amounts of BES (2-bromoethanesulfonate, A type of methanogenic inhibitor) were added into the mono- and co-culture systems in order to investigate the mechanism by which Methanogen 166 promotes caproic acid production in the binary caproic acid fermentation system. All incubations were conducted in triplicate, and presented results represent the mean values of three independent experiments.

### Optimization of co-culture fermentation parameters

For optimization studies, the caproic acid co-culture fermentation was carried out anaerobically as described above over a period of 9 days. Samples were taken at the end of this fermentation period for analysis and comparison. The effect of the inoculum size on caproic acid production was studied by utilizing different inoculum sizes (5%, 10%, 15%, and 20% v/v) of 7-day old cultures, as well as different ratios (2:1, 1:1, 1:2, v/v) of *C. kluyveri* H588 and Methanogen 166 prepared in the MB medium.

Additionally, in order to investigate the influence of initial acetate, ethanol, and yeast extract concentrations on resultant caproic acid production in this binary fermentation system, different quantities of each substance were added to MB media following the optimization of *C. kluyveri* H588 and Methanogen 166 inoculation size and ratios. All experiments were performed in triplicate, and the results presented are the average of three trials.

### Inoculum preparation for pilot-scale fermentation

The seed cultures for the pilot-scale fermentation system were prepared as follows. For *C. kluyveri* H588, a 700 mL serum bottle containing 540 mL of the MB medium (for the second-stage culture) was inoculated with 60 mL of the liquid first-stage culture of *C. kluyveri* H588, and incubated anaerobically for 7 days at 35 °C. The third-stage culture was then prepared by inoculating 600 mL of this second-stage culture into a 7 L anaerobic bottle containing 5400 mL MB medium which was incubated for 7 days as above, after which the entire volume of this bottle was used to inoculate a 70 L fermenter (v/v, 10%). After an appropriate incubation period that allowed these bacteria to enter the middle of their exponential growth phase, this fourth-stage seed was used for the pilot-scale fermentation.

For Methanogen 166, seed cultures were prepared in four stages as above, except that strict anaerobic conditions were maintained and the final seed culture volume was 3 L. At each stage, the gas phase of the bottle or fermenter contained a mixture of hydrogen (80%, v/v) and carbon dioxide (20%, v/v), and this mono-culture was incubated anaerobically in MC medium at 35 °C for 7 days, at which point the exponential growth phase had been reached.

### Pilot-scale batch co-culture fermentation

Pilot-scale experiments were conducted in a pilot-scale plant built at Anhui Yingjia Distillery Co., Ltd., Luan City, People’s Republic of China under conditions meant to simulate a more typical industrial environment in order to validate the results of lab-scale experiments. In this pilot-scale batch fermentation study, a 1000 L anaerobic caproic acid co-culture fermentation reactor with an effective volume of 900 L was used. The pilot-scale fermentation was conducted using the optimized caproic acid production conditions identified in the laboratory for this binary co-culture system. A schematic diagram of the experimental apparatus used in this study is shown in Additional file 1.

The pilot-scale system was composed of five functional parts: a nitrogen storage tank, an inoculum preparation system, a mixing tank, a co-culture fermentation system, and a storage tank. All components were made of stainless steel.

Prior to initiating pilot-scale fermentation, the fermenter was subjected to a thorough manual cleaning followed by sterilization-in-place. Sterilization was carried out using low pressure steam to heat containers and transfer lines, and steam condensate was drained from low points through steam traps. Transfer lines were held at 121 °C for at least 30 min, and the adequacy of pipe surface temperatures was verified using a thermometer. All empty containers except the mixing tank and their associated housings were sterilized prior to use via heating to 121 °C and holding there for 30 min. The fermentation medium was prepared by mixing all media components except for ethanol and CaCO_3_ in the mixing tank. Then this mixed liquid was sterilized using a plate heat exchanger and was subsequently pumped into the caproic acid fermenter. Next, the temperature of the medium was adjusted to 35 °C by applying cold tap water to the exterior of the bioreactor. Subsequently, the bioreactors and the associated transfer lines were kept under anaerobic conditions by continuous sparging of nitrogen gas for 10 min, and then the mixed liquid was combined with then ethanol and CaCO_3_, and was inoculated with the seed cultures of *C. kluyveri* H588 and Methanogen 166. Pressure inside the fermenter was initially set to 100–200 mbar, and the gas produced was released through a relief valve which opened at a fixed pressure of 200 mbar during fermentation. Caproic acid content was monitored at various time intervals. After fermentation, the broth was pumped into the storage tank and used for pit-entry fermentation.

### Pit-entry fermentation

Pit-entry fermentation experiments were performed using three industrial traditional fermenting pits. Each pit was a rectangular underground pool constructed of pit mud, a specific fermented clay that provides a suitable habitat for the brewing microorganisms. For each pit, the top length and width were 3600 mm and 2300 mm, respectively, the bottom length and width were 2800 mm and 1540 mm respectively, and the depth was 2400 mm. Three additional pits with the same volume were used as controls. All pits were built in 2012.

In accordance with the brewing process of traditional Chinese strong flavor liquor (Yan et al. 2015a, b), raw materials (sorghum, rice, glutinous, rice, maize, and wheat) were crushed into pieces and splashed with hot water (~ 70 °C) at a ratio of 1:0.25. After approximately 1 h, the wet mixed grains were further mixed with steamed rice husks and the fermented *Zaopei* obtained from the previous stage of fermentation. Then this mixture was transferred into a distillatory for liquor distillation. After distillation, the multiple-grain *Zaopei* was mixed with starter Chinese *Daqu* as a process-defined ratio, placed into pits, covered by pit mud, and subjected to fermentation. Control pits were then fermented using natural conditions for 70 days. For the experimental pits, part of the upper pit mud was moved and 500 L of the co-culture fermentation broth was added into the *Zaopei* from the top to the bottom after ethanol fermentation was almost complete (~ 20 days in this study). Experimental pits were then sealed with pit mud and again subjected to fermentation for another 50 days. After fermentation, the fermented *Zaopei* from the test and control pits was collected and distilled with steam to extract the ethanol and other flavour compounds. The quality of each raw liquor was then evaluated as a means of assessing the various pit-entry fermentation conditions.

### Analytical methods

A Perroff-Hausser counting chamber was used to count both microbes under an oil immersion lens with dark-field microscopy conditions and 0.1% Loeffler’s Methylene Blue Stain Solution as a contrast dye. Concentrations of caproic acid and butyric acid in the fermented broth, as well as levels of the primary aromatic compounds in the distilled liquor were measured via Agilent 7890 gas chromatography with a HP-IN-NOWax capillary column (30 m × 0.32 mm i.d. and 0.25 μm film thickness) and a flame ionization detector (FID). The chromatogram was run at a 180 °C oven temperature and a 90 °C injection temperature using N_2_ as a carrier gas and H_2_ as a flaming gas (Yan et al. 2015a, b). The fermented broth was first filtered through a 0.22 μm filter prior to being subjected to a Gas Chromatograph equipped for measuring caproic acid concentrations. H_2_ and CH_4_ contents were measured via Gas Chromatograph (Shimadzu GC2014, Japan) using a thermal conductivity detector and a ShinCarbon St 80/100 column with helium gas as a carrier gas as previously reported by Yang et al. (2002). The GC operating conditions were as follows: injection temperature, 100 °C; flow rate, 10 mL/min; column temperature, 40 °C for 3 min, then increased to 140 °C at 25 °C/min and held at 140 °C for 3.5 min. The TCD temperature was held constant at 200 °C. Gas sampling and injections were made with a Hamilton pressure lock syringe. The sensory characteristics of taste, smell, aftertaste, acidity, and overall acceptability for each final distilled liquor was evaluated by 10 well-trained experts working in the Anhui Yingjia Distillery Co., Ltd., Luan, China. Among these, 4 experts were females of 30–40 years old and 4 were 28–45 years old. The sensory scores were determined using a 9-point hedonic scale (1, dislike extremely; 2, dislike very much; 3, dislike moderately; 4, dislike slightly; 5, neither like nor dislike; 6, like slightly; 7, like moderately; 8, like very much; 9, like extremely). The mean intensity scores for all attributes were calculated and plotted.

## Results

### Caproic acid fermentation using single and mixed cultures

**Fig. 1:**
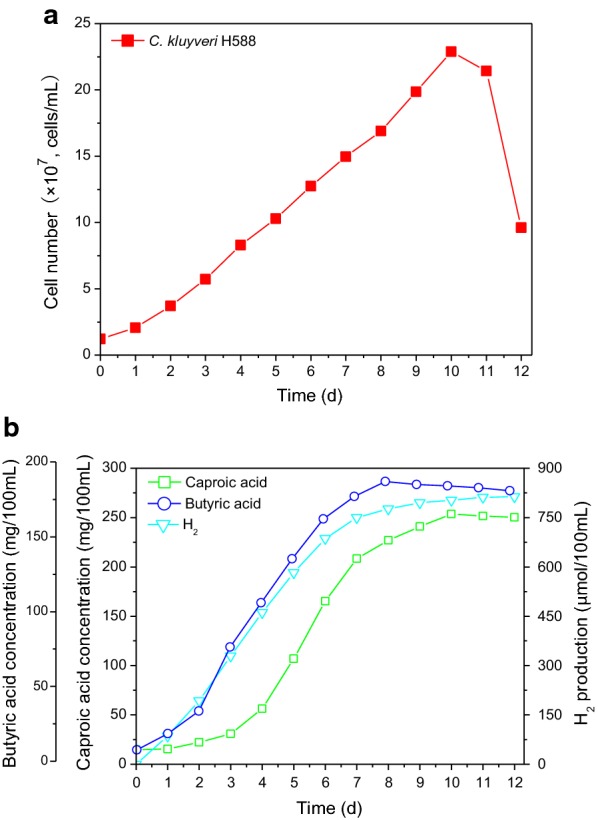
Time courses of cell growth (**a**), caproic acid, butyric acid and H_2_ production (**b**) for the mono-culture fermentation system

**Fig. 2:**
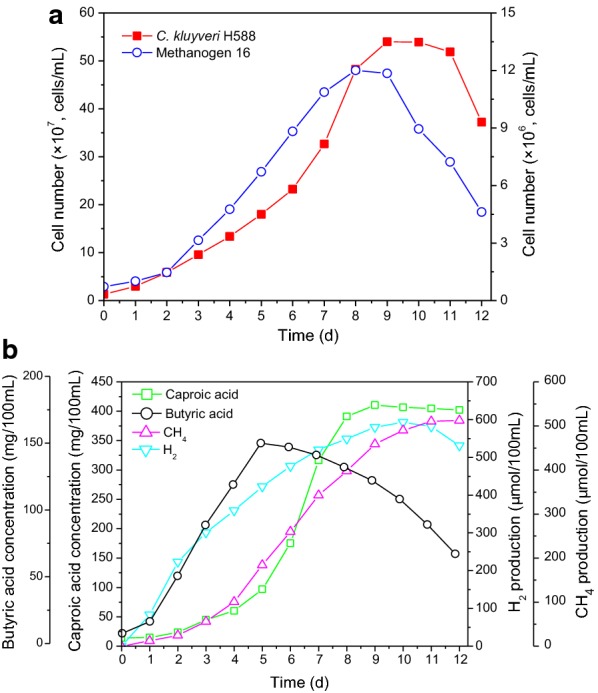
Time courses of cell growth (**a**), caproic acid, butyric acid, H_2_ and CH_4_ production (**b**) for the co-culture fermentation system

The growth dynamics of the *C. Kluyveri* H588 mono-culture fermentation system and the combined *C. Kluyveri* H588 and Methanogen 166 co-culture fermentation system are shown in Figs. 1a and 2a. *C. Kluyveri* H588 cell numbers increased over time and reached a peak value of 2.29 × 10^8^ cells/mL by day 10 in the mono-culture fermentation setting. Cell numbers remaining constant until day 11, after which extensive sporulation was observed and cell number rapidly decreased to 9.61 × 10^7^ cells/mL by day 12. The observed sporulation was likely due to unfavorable growth conditions that arose during fermentation. In the co-culture fermentation system, *C. kluyveri* H588 cell numbers grew from 1.31 × 10^7^ to 3.26 × 10^8^ cells/mL in a similar pattern to that observed in the mono-culture system during the initial 7 days of fermentation. After that time growth continued until reaching a maximum after 9 days of fermentation. Growth then entered a stationary phase until day 11, after which cell numbers declined.

Figure 1a illustrates the fact that the maximum *C. kluyveri* H588 cell number obtained in the co-culture system was higher than that achieved during mono-culture. These results demonstrated that *C. kluyveri* H588 achieved a superior biomass in the MB medium when co-cultured with Methanogen 166, suggesting that Methanogen 166 had a significant influence on the growth of *C. kluyveri* H588. The same phenomenon is consistent with other reports regarding mixed culture fermentations using ruminal cellulolytic bacteria and *C. Kluyveri* (Kenealy et al. 1995).

The influence of the co-culture of *C. kluyveri* H588 and Methanogen 166 on the production of caproic acid, butyric acid, H_2_, and CH_4_ during fermentation is shown in Fig. 2b. Butyric acid levels first increased and then decreased in the co-culture fermentation system, which differed from what was observed in the mono-culture fermentation in which butyric acid levels increased over the first 8 days and then stabilized, although maximal butyric acid levels were lower at all times in this system. Caproic acid began to accumulate only after 3 days in both the mono- and co-culture fermentation systems. Typically caproic acid is produced secondary to the formation of butyric acid, as butyric acid is a precursor for caproic acid production. In present study, *C. kluyveri* H588 mono-culture led to a maximum caproic acid concentration of 253.69 mg/100 mL, with the entire fermentation process being completed in 10 days. In contrast, the co-culture fermentation process required only 9 days and yielded greater caproic acid concentrations of 410.21 mg/100 mL. Compared with the mono-culture fermentation system, the accumulation of caproic acid in the mixed culture fermentation containing both *C. kluyveri* H588 and Methanogen 166 was therefore increased by 61.7%. This indicates that co-culture with both strains not only significantly improved caproic acid production, but also shortened the peak production period.

In the mono-culture system, H_2_ production reached a maximum of 802.57 μmol per 100 mL of culture broth after 10 days of cultivation, with slight fluctuations and a minor overall increase by the end of fermentation (Fig. 1b). In contrast, when *C. kluyveri* H588 was co-cultured with Methanogen 166 the levels of H_2_ remained low, whereas CH_4_ levels were observed to gradually increase throughout fermentation. The above result made it clear that the co-culture system resulted in significantly increased caproic acid production than the mono-culture system, suggesting an important role for Methanogen 166 in this process. We confirmed that in the mixed cultures of *C. kluyveri* and Methanogen, *C. Kluyveri* contributed hydrogen which Methanogen 166 was then able to utilize for the production of CH_4_. At the same time, the consumption of hydrogen by Methanogen 166 alleviated the feedback inhibition of hydrogen on *C. kluyveri* H588, thereby improving caproic acid production (Hu et al. 2005). This mutual relationship has been found to be particularly evident in the pit mud of strong aroma Chinese liquor, as both *C. Kluyveri* and Methanogen 166 co-exist in the pit mud where they form a symbiotic relationship with each other. Previous research has shown that Methanogen 166 is present at particularly high levels in aged pit mud (Ding et al. 2014), normally towards the bottom section of the pit with comparably low levels in upper regions. In the traditional brewing of Chinese strong flavor liquor, high quality raw liquor is normally obtained from the bottom layers of *Zaopei*, which may be due to the larger amounts of Methanogen present in this region, allowing for superior elimination of hydrogen inhibition during the fermentation process, thus enhancing the production of aromatic compounds. Similar results have also been reported by Zhang et al. (2013).

### The effects of BES on single and mixed culture fermentation

**Fig. 3:**
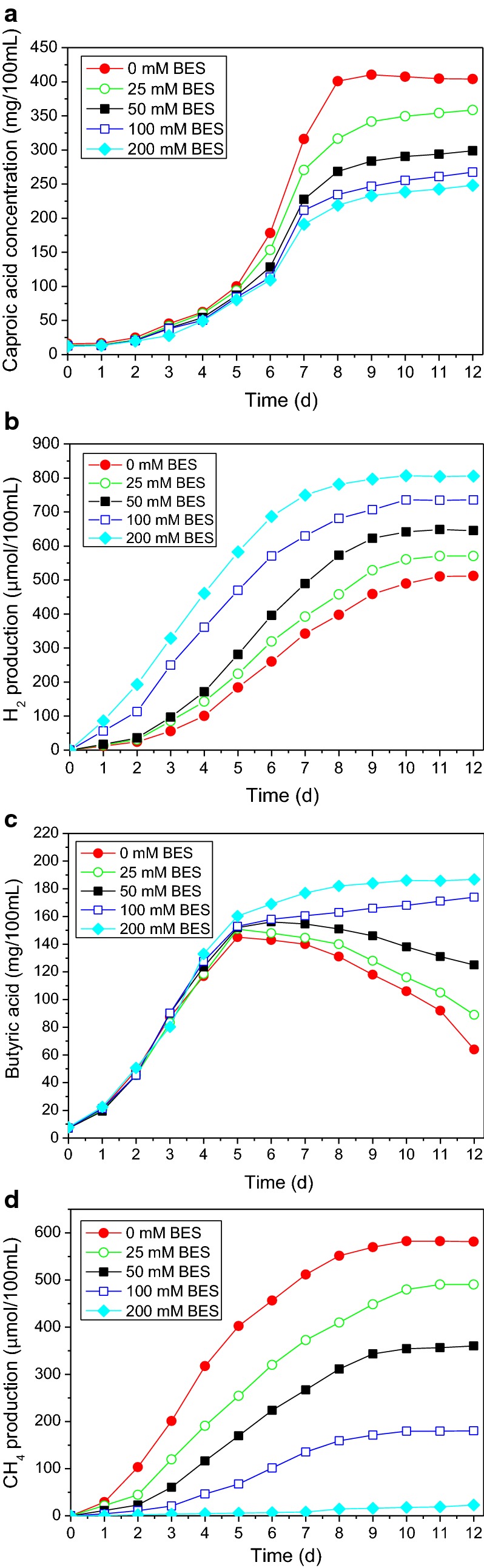
Accumulation of caproic acid (**a**), butyric acid (**b**), H_2_ (**c**) and CH_4_ (**d**) in the mixed-culture fermentation system of *C. kluyveri* H588 and Methanogen 166 under different BES concentrations

During the co-culture fermentation of *C. kluyveri* H588 and Methanogen 166 without the addition of a methanogenic inhibitor, caproic acid and methane accumulated steadily, whereas hydrogen and butyric acid accumulated initially and then later rapidly decreased to constant low levels (Fig. 3). In the groups to which BES was added, in contrast, caproic acid levels decreased as BES concentrations increased, with a similar trend observed for methane production. Methane accumulation was strongly inhibited at BES concentrations of 100–200 mM, while it was more moderately inhibited at concentrations under 25–50 mM. As a classic structural analogue of coenzyme M, BES is a commonly used methanogen-specific inhibitor (Nollet et al. 1997) in microbiological research (Bradley and Chapelle 2000; Scholten et al. 2000; Liu et al. 2011). However, the effective inhibitory concentrations of this compound differ situationally. For example, Conrad and Klose (2000) reported that 10 mM BES was the optimal concentration necessary to inhibit anaerobic methanogens present in rice roots systems. There are also reports about that in a thermophilic anaerobic digester, the complete inhibition of the methanogenesis required at least 50 mM BES, with even higher BES concentrations being needed for the inhibition of hydrogenotrophic methanogens than for acetoclastic methanogens (Zinder et al. 1984). In the present binary co-culture fermentation system, the production of methane appears to be blocked almost completely (relative production < 5% compared to that of the control) at BES concentrations over 200 mM.

Figure 3 also illustrates that the concentrations of H_2_ increased as BES concentrations increased, indicating that Methanogen 166 utilization of H_2_ was compromised following enhanced BES inhibition. This further suggested that this accumulated hydrogen can directly drive the stimulation of butyric acid production. Our results are consistent with the observations of Xu et al. (2010) who reported an accumulation of hydrogen in a mesophilic sludge digester after the addition of BES, suggesting effective inhibition of methane accumulation.

**Fig. 4:**
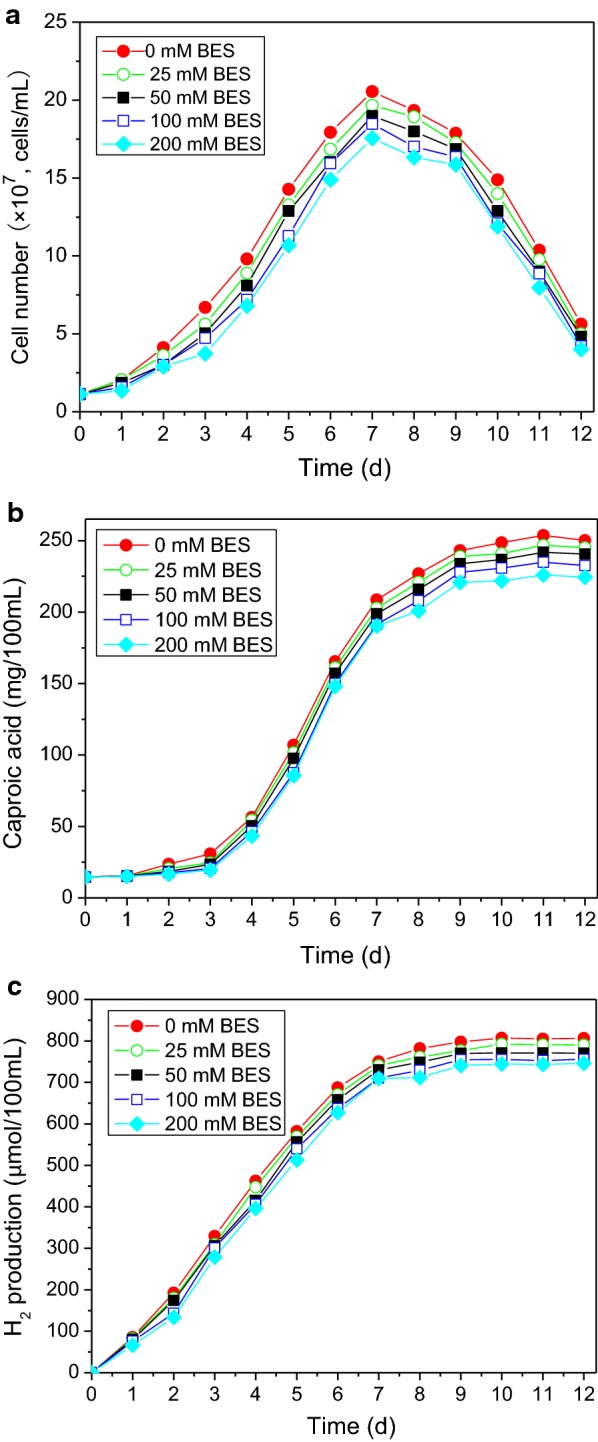
Accumulation of cell number (**a**), caproic acid (**b**) and H_2_ (**c**) in the single culture fermentation of *C. kluyveri* H588 under different BES concentrations

The effects of BES on the single culture fermentation of *C. kluyveri* H588 are presented in Fig. 4. BES had no substantial effect on cell growth or production of caproic acid or H_2_ by *C. kluyveri* H588 at concentrations as high as 200 mM. This further supports the hypothesis that in our co-culture system BES acts by inhibiting methanogenesis directly, without affecting the activity of other microbes present except for those capable of reducing dechlorinate polychlorinated biphenyls and chlorinated ethanes (Chiu and Lee 2001). We have thus shown BES to be an effective inhibitor capable of suppressing methanogenic activity without directly inhibiting *C. kluyveri* H588. Therefore, BES can be used as a means of exploring the binary system fermentation mechanisms underlying our *C. kluyveri* H588 and Methanogen 166 co-culture.

By comparing the levels of caproic acid, butyric acid, H_2_, and CH_4_ present during single and the mixed culture fermentations, we can conclude that BES caused a strong inhibition of methane production and induced the accumulation of H_2_ and butyric acid (Figs. 3, 4). Figure 4 also shows that the production of caproic acid is significantly negatively correlated with the H_2_ production. Wu (2000) demonstrated that high levels of H_2_ produced from caproic acid fermentation by *C. Kluyveri* can act in a feedback loop to inhibit the growth and metabolism of *C. Kluyveri*, decreasing subsequent caproic acid production. However, when *C. kluyveri* was co-cultured with a Methanogen, this caproic acid fermentation could be regulated via “interspecies hydrogen transfer”, wherein the Methanogen converts the accumulated hydrogen into methane, thereby eliminating the product inhibition of hydrogen on *C. kluyveri*. Our study supports this finding.

### Optimization of co-culture fermentation parameters

Our work thus far demonstrates that *C. kluyveri* H588 and Methanogen 166 act in a symbiotic relationship, cooperating to enhance caproic acid production in the binary co-culture fermentation system. However, they also competed for nutrients such as sodium acetate as they grow and metabolize. Therefore, it is necessary to ease this competition and strengthen cooperation as much as possible by optimizing certain important culture conditions during the fermentation process. In the following experiments, the effects of inoculation size, acetate, ethanol, and yeast extract concentrations on this co-culture fermentation system were investigated in order to explore their influence on caproic acid production.

### Effects of inoculation sizes on co-culture fermentation

The effect of mixed inoculum sizes with different ratios of *C. kluyveri* H588 and Methanogen 166 on the caproic acid accumulation are detailed in Table 1. Caproic acid production by the binary fermentation system increased as the overall combined inoculation size increased until a maximum of 10%, after which point further increases adversely impacted caproic acid production. Maximum caproic acid production (435.72 ± 13.58 mg/L) was obtained via the fermentation with a mixed inoculum size of 10% and a *C. kluyveri* H588/Methanogen 166 inoculation ratio of 2:1 (v/v). Further increases or decreases in the size and ratio of this inoculum decreased caproic acid production.

**Table 1:** Effect of different inoculum sizes and ratios of *C. kluyveri* H068 and *Methanogen* 166 on the production of caproic acid after 10 days of co-culture fermentation

Inoculum size (%)	*C. kluyveri* H068:*Methanogen* 166 (v/v)	Caproic acid (mg/100 mL)
	2:1	379.83 ± 13.65
5	1:1	344.28 ± 10.84
	1:2	323.59 ± 11.76
	2:1	435.72 ± 13.58
10	1:1	407.56 ± 14.45
	1:2	385.45 ± 13.15
	2:1	410.68 ± 12.81
15	1:1	387.47 ± 13.34
	1:2	364.2 ± 12.54
	2:1	390.85 ± 11.12
20	1:1	363.87 ± 10.54
	1:2	346.43 ± 8.32

The inoculum size is an important parameter that influences both the specific growth rate and fermentation time. Generally, a small inoculum size requires a longer period of time to achieve fermentation, whereas large inoculum sizes lead to a rapid increase in biomass and thereby decrease fermentation time. However, extremely large inoculum sizes can also decrease the production yield, potentially due to the overuse of substrate for growth and maintenance at high cell concentrations. Our study confirmed this finding, with caproic acid production decreasing if the initial combined inoculum size was greater than 10%. In this study, there was a significant difference in caproic acid production between a 10% inoculum containing a mix of *C. kluyveri* H588/Methanogen 166 at a ratio of 2:1 and all other tested sizes and ratios (Table 1). Thus, this inoculum size was adopted for use in our subsequent studies.

### Effects of sodium acetate and ethanol on co-culture fermentation

**Fig. 5:**
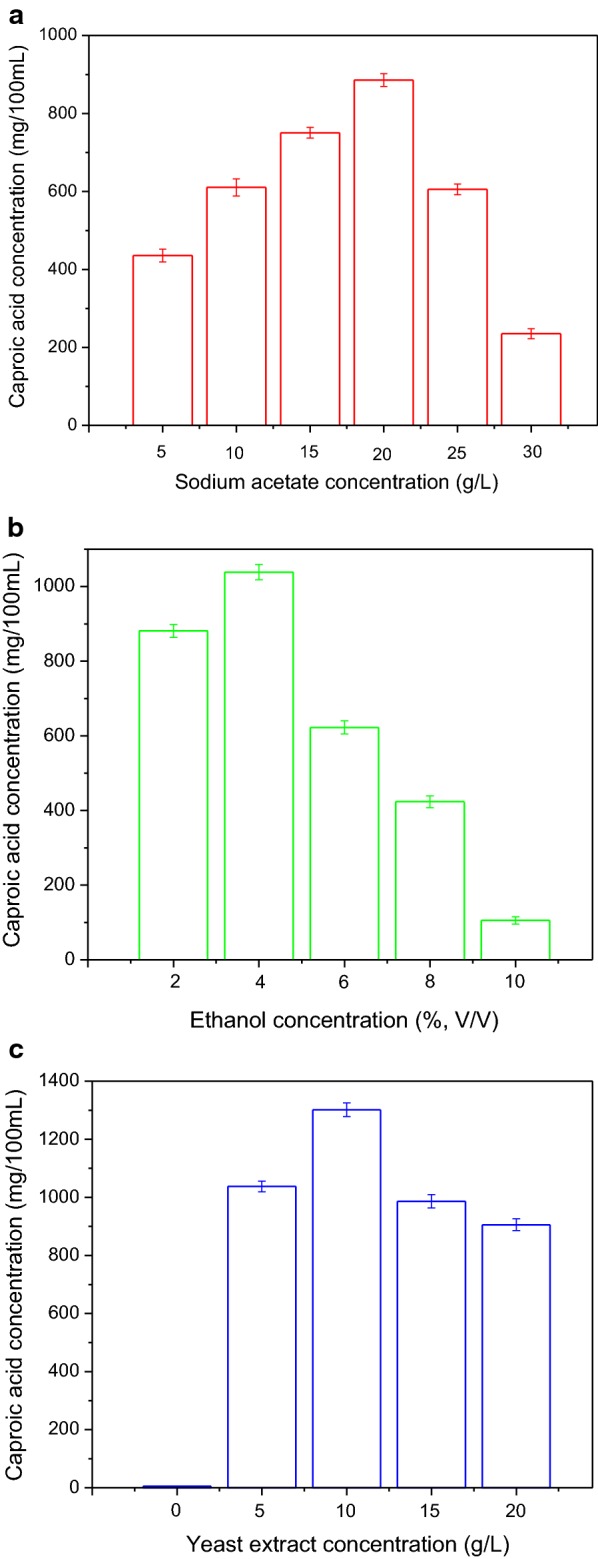
Effect of different initial sodium acetate concentrations (**a**), ethanol concentrations (**b**), and yeast extract concentrations (**c**) on caproic acid production by the co-culture fermentation of *C. kluyveri* H588 and Methanogen 166

Sodium acetate is one of the important nutrients required for *C. Kluyveri* to grow and produce caproic acid. The influence of different initial sodium acetate concentrations on caproic acid production in our co-culture fermentation system is illustrated in Fig. 5a. The production of caproic acid increased with as sodium acetate levels increased, with maximal caproic acid levels of 885.67 ± 16.27 mg/100 mL being obtained at an initial sodium acetate concentration of 20 g/L. At sodium acetate concentrations greater than 20 g/L, caproic acid concentrations sharply decreased, potentially due to the adverse effects of the high osmotic pressure introduced at high sodium acetate concentrations, which inhibits the activity of *C. kluyveri* H588 and Methanogen 166. Therefore, in order to achieve high caproic acid fermentation efficiency, the optimum initial sodium acetate concentration should be 20 g/L.

Ethanol is the main product during the traditional brewing of Chinese strong flavor liquor, and it is also one of key factors influencing caproic acid production. In the present study, various amounts of ethanol were added into our binary fermentation system to investigate its effects on caproic acid production. As illustrated in Fig. 5b, caproic acid concentrations increased with the addition of ethanol, and a maximum concentration of 1038.56 ± 32.46 mg/100 mL was obtained at a 4% (v/v) ethanol concentration. At ethanol levels greater than 4% (v/v), caproic acid production decreased. Microscopic examination also revealed that the *C. kluyveri* H588 strain grew best under a 4% (v/v) ethanol concentration, growing poorly in the presence of higher levels of ethanol. In particular, ethanol levels greater than 6% (v/v) caused *C. kluyveri* H588 to become thin and produce spores (data not shown).

The above results suggest that the growth and the metabolism of *C. Kluyveri* was inhibited as ethanol concentrations greater than 4% (v/v), as previously reported by Yao and Zhang (2008). In the traditional brewing industry, pit mud is normally maintained via sprinkling with tail wine. Although tail wine contains substantial nutrients that can be transformed into flavour compounds during the brewing process, it can substantially inhibit the activity of *C. Kluyveri* as it contains an ethanol concentration of greater than 10% (v/v), and the long-term use of tail wine was thus shown to cause pit mud to age quickly. Therefore, several measures have been adopted by Chinese researchers to maintain the pit mud. For example, instead of tail wine researchers use pure *C. kluyveri* cultures, as this is a key functional microorganism present in pit mud. In addition, diluted “yellow water” or other nutritional liquids is instead added to these pits, with one or more of these approaches being employed simultaneously.

Acetate and ethanol are the two major carbon sources required for the growth and metabolism of caproic acid producing bacteria. During the solid state fermentation of Chinese strong flavor liquor, starches present in the multiple-grain *Zaopei* are initially saccharified to glucose, before subsequently being transformed into ethanol by yeast. After ethanol production, fermentation gradually enters into an acid-producing stage during which some of the ethanol is transformed into acetic acid. This ethanol and acetic acid are then used as precursors by *C. Kluyveri* for caproic acid production. During this process of traditional liquor brewing, high quality *Daqu* containing a variety of microorganisms, such as *Bacillus*, *Acetobacter*, *Aspergillus*, *Mucor*, *Saccharomyces* and *Hansenula,* as well as various enzymes including amylase, glucoamylase, and protease, must be used to produce ethanol and acidic substances, further enhancing the growth and metabolism of caproic acid bacteria. During the actual production process, the ratio of grains to fermented *Zaopei* should be adjusted based on the specific brewing technology being utilized. And in most cases, the initial *Zaopei* starch concentration should be between 15 and 22%, which will allow it to meet the carbon requirements of the brewing microorganisms (Yan et al. 2015a, b).

### Effects of yeast extract on co-culture fermentation

Yeast extract is the common name for various forms of processed yeast products made by extracting yeast cell contents via cell wall removal. Its major constituents are protein, amino acids, peptides, nucleotides, B vitamins, and trace elements. Yeast extract has been used primarily as either a food additive or flavouring, or as a nutrient source for microbial culture media.

It has been reported that yeast extract is an ideal nitrogen source for caproic acid fermentation, as it not only rich in nitrogenous compounds, but also supplies growth factors for the caproic acid bacteria (Bornstein and Barker 1991). In present study, a series of batch co-culture fermentations were performed using media supplemented with yeast extract at concentrations ranging from 0 to 20 g/L, and the results are shown in Fig. 5c.

As shown in Fig. 5c, without the addition of yeast extract to the fermentation medium, no caproic acid was produced. This is likely because some growth factors (such as biotin) present in the yeast extract were indispensable for *C. kluyveri* H588 metabolism (Bornstein and Barker 1991). Caproic acid production increased as yeast extract levels increased, and the maximum concentration of caproic acid (1301.62 ± 23.08 mg/100 mL) was obtained at the yeast extract concentration of 10 g/L. Further increases in yeast extract did not enhance caproic acid production, potentially due to an enhancement in bacterial growth at the cost of caproic acid production at higher concentrations.

Nitrogen sources are indispensable for maintaining the growth and replication of *C. Kluyveri*, and while this strain can utilize some organic nitrogen efficiently, it is unable to grow well in media suitable for many *Clostridium species*; milk, peptone, tryptone and glucose, for example, are all inadequate substrates. Normally, fairly good growth has been achieved only in media containing an extraordinarily high concentration of yeast extract and some ethanol without any effect from the further addition of peptone, tryptone, or glucose. During the traditional fermentation of Chinese strong flavor liquor, nitrogen primarily comes from the *Daqu* powder and from the grains and dead microbial cell proteins present in the *Zaopei* and pit mud. Typically the levels of nitrogen are not overly high, as larger quantities can lead to the production of fusel oils during fermentation, which can have undesirable effects upon consumption. Therefore, high-protein raw grain materials are generally not chosen to be used in an industrial brewing setting.

### Scaling-up and application to strong aroma Chinese liquor brewing

**Fig. 6:**
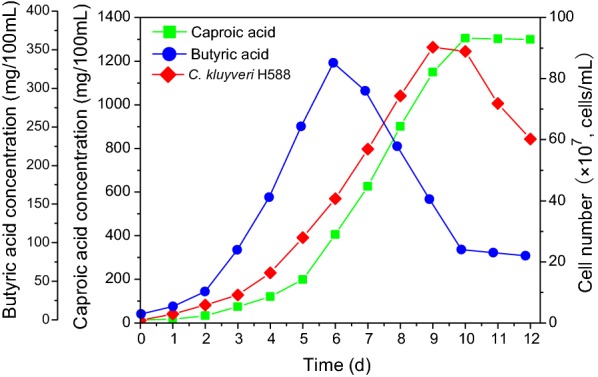
Time courses of caproic acid production, butyric acid production and cell growth of *C. kluyveri* H588 during binary fermentation system carried out in pilot reactors

In order to identify problems that were not significant at the laboratory scale used in our earlier experiments, and also to determine whether fermentation yields are maintained when working with larger volumes, it is necessary to conduct pilot-scale experiments under more realistic conditions. In present study, the binary *C. kluyveri* H588 and Methanogen 166 co-culture fermentation system was scaled up in a 1000 L fermenter, and batch fermentations were carried out to test the ideal parameters identified in the laboratory setting. Changes in cell growth and production of caproic acid and butyric acid over time in the 1000 L bioreactor are illustrated in Fig. 6. After a 3 day lag period, *C. kluyveri* H588 grew well in this co-culture system, achieving a maximum cell number of 9.03 × 10^8^ cells/mL at day 9 of fermentation. The growth curve of this strain was of a logistic-shape, with a lag phase (day 0–3), a log phase (day 3–9), and a stationary phase after day 9. After this stationary phase, *C. kluyveri* H588 cell counts rapidly declined until the end of fermentation. This sharp decrease in cell numbers from day 10–12 may have been caused by the inhibitory effects of caproic acid accumulation, and/or by nutrient depletion. These results were similar to those obtained in the laboratory setting.

Figure 6 also illustrates the variations of butyric acid and caproic acid over time in the co-culture system. The general change trends of these two acids were identical to those obtained in the lab-scale experiments. Butyric acid levels slightly increased over the first 2 days of fermentation, and then increased dramatically and reached a maximum of 332.85 mg/100 mL at day 6. This concentration then dropped sharply until day 10, and subsequently remained constant until to the end of fermentation. There was no significant change in caproic acid during the initial 2 days. Caproic acid production then rapidly increased from day 3–10, reaching a maximum concentration of 1305.56 mg/100 mL, after which it was largely unchanged. As compared with the lab-scale shake flask fermentation experiment, this pilot-scale fermentation reaction was faster and achieved a higher cell concentration, potentially due to differences in the geometry of each system.

Once the pilot-scale co-culture fermentation entered into the stationary stage, the culture broth was collected and used for pit-entry fermentation. After about 50 days, the fermented *Zaopei* from the experimental and control pits was collected, and each was distilled using low pressure steam to extract ethanol and other flavour compounds according to traditional Chinese liquor distilling methods. In the present study, the crude liquor distilled from each pit of *Zaopei* was collected separately, and its quality was used as the criterion for the assessment of this pit-entry experiment. Each result represented the average value of the corresponding data obtained from the experimental and control pits, respectively. Table 2 lists the concentrations of volatile flavour substances in the crude liquor distilled from the fermented *Zaopei* in the experimental and control pits, respectively. Table 2 revealed that the maximum ethyl caproate concentration (286.29 ± 9.82 mg/100 mL) was obtained from the distillation of the experimental *Zaopei*, and that this concentration was 142.78% higher than that obtained from the control pits. In addition, higher levels of ethyl acetate and ethyl butyrate were present in the crude liquor following pit-entry treatment. Conversely, levels of ethyl lactate in the control pit were higher.

**Table 2:** The concentrations of flavour compounds in the raw liquor obtained from the fermented Zaopei of experimental and control pits, respectively (mg/100 mL)

Compounds	Control (mg/100 mL)	Pit-entry fermentation (mg/100 mL)
Ethyl acetate	75.36 ± 3.12	118.4 ± 4.68
Ethyl caproate	117.92 ± 10.94	286.29 ± 9.82
Ethyl butyrate	47.77 ± 6.32	81.4 ± 3.74
Ethyl lactate	283.73 ± 12.1	101.32 ± 5.62
Acetic acid	39.31 ± 3.58	48.83 ± 2.13
Caproic acid	15.94 ± 0.92	34.22 ± 2.67
Butyric acid	14.62 ± 0.52	18.71 ± 0.98
1-Propanol	13.2 ± 0.87	19.89 ± 1.18
Isobutanol	8.38 ± 0.76	11.05 ± 0.79

## Discussion

This study developed a process for producing caproic acid using a co-culture fermentation system utilizing both *C. kluyveri* H588 and Methanogen 166, with the results revealing that a symbiotic relationship exists between these two bacterial strains. Although *C. Kluyveri* can grow and produce caproic acid alone under anaerobic conditions, the cell numbers and levels of caproic acid were significantly lower than those obtained from mixed co-culture with Methanogen 166. These two species have been shown to interact in a mutually beneficial manner via “interspecies hydrogen transfer” (Ding et al. 2014). The stability of this binary fermentation system depends upon the concentration of hydrogen. An increase in hydrogen inhibits the growth of the H_2_-producing *C. Kluyveri* species, but stimulates the growth of H_2_-consuming methanogenic species. The results of the present study indicate a tight coupling of hydrogen transfer between *C. Kluyveri* and Methanogen 166.

**Fig. 7:**
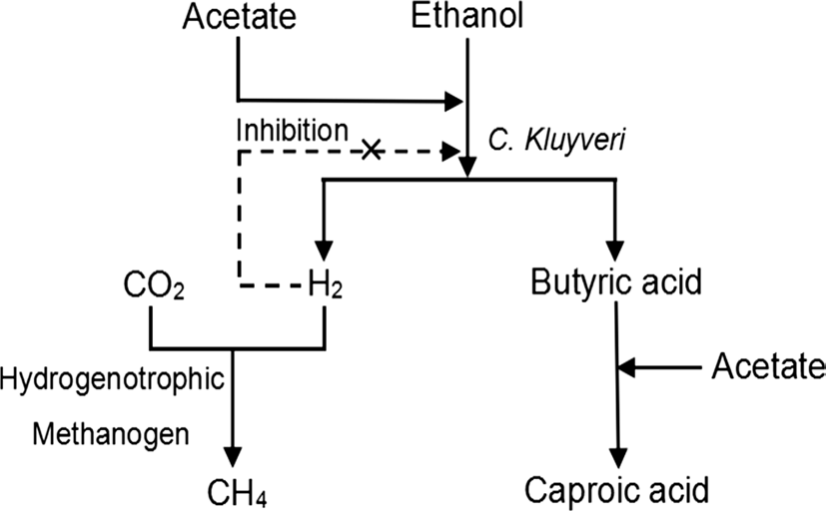
The synthesis metabolic frame of caproic acid during the co-culture fermentation

The metabolic synthesis of caproic acid during co-culture fermentation as previously suggested (Hu et al. 2005), is shown in Fig. 7. Both acetate and ethanol were metabolized by *C. kluyveri* H588 to produce butyric acid and molecular hydrogen. This butyric acid then associated with ethanol and was converted to caproic acid. Butyric acid and hydrogen are both intermediates in the synthesis of caproic acid. Normally, high levels of butyric acid help promote the production of caproic acid, whereas high hydrogen levels cause the feedback inhibition to *C. Kluyveri* metabolic activity (Wu 2000). In mixed cultures, Methanogen 166 uses hydrogen as an electron donor to produce methane. Thus, decreasing hydrogen levels promote the accumulation of caproic acid. Our study also revealed that methane can also be produced by a Methanogen 166 mono-culture in MB medium (data not shown). This suggests that methane is not only formed via the hydrogenotrophic pathway, but also via the aceticlastic pathway. Therefore, a competition for acetate between these co-cultured microbes also exists.

In the traditional brewing of Chinese strong flavor liquor, the methanogenic species present in pit mud, coupled with caproic acid-producing microorganisms, were the dominant sources of the main flavour compounds of the resultant liquor. Their interactions, mutual influence, and coordinated development constitute the specific microecological system of the pit (Yan et al. 2015a, b). Methane fermentation promotes caproic acid fermentation in order to produce additional caproic acid, and it further helps to increase the accumulation of ethyl caproate, which eventually enhances the quality and style of Chinese strong flavor liquor. Generally, the Chinese liquor industry refers to the commensal species of caproic acid producing bacteria and methanogens as “the cellar mud caproic acid bacteria”, rather than naming the strains individually.

Earlier studies have suggested that ethyl caproate, ethyl acetate, ethyl butyrate, and ethyl lactate are the four major volatile flavour components present in Chinese strong flavor liquor, with their relative proportions strongly influencing the aromatic quality and style of the spirit. Among these esters, ethyl caproate is the major component most important for the aroma and bouquet of the liquor. Normally, high ethyl caproate concentrations are associated with higher quality Chinese strong flavor liquor, whereas high ethyl lactate levels can make the liquor taste bitter, stuffy, and lacking in fragrance, thereby disrupting the typical character of Chinese strong flavor liquor (Bornstein and Barker 1991). Therefore, it is important to improve the production of caproic acid in the Chinese strong flavor liquor industry. In this study, the binary fermentation conditions of *C. kluyveri* H588 and Methanogen were optimized, and these optimized conditions were scaled-up to use in a 1000 L pilot-scale fermenter. The resultant caproic acid broth was subjected to traditional pit-entry fermentation in an attempt to improve the quality of Chinese strong flavor liquor. Table 2 revealed that the maximum concentration of caproic acid (286.29 ± 9.82 mg/100 mL) was obtained from the distillation of the experimental *Zaopei*; a concentration 142.78% higher than that obtained from control pits.

**Fig. 8:**
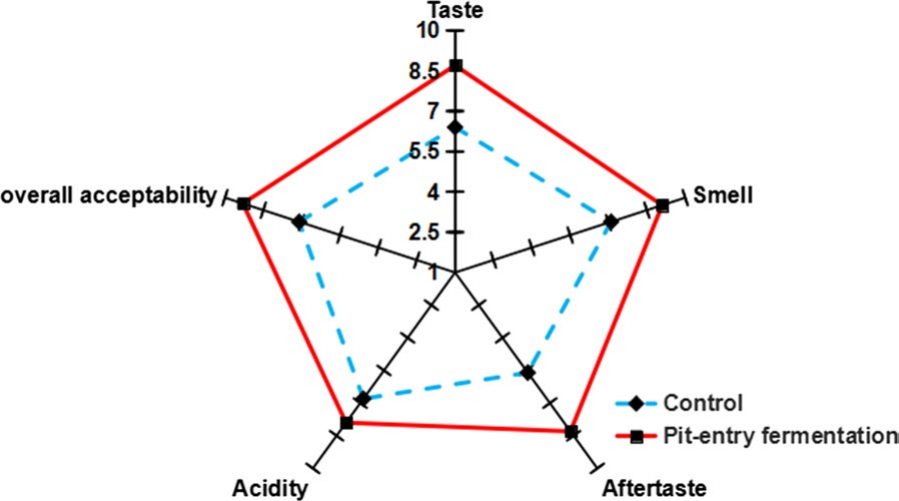
The sensory profiles of the raw liquors obtained from the fermented *Zaopei* of the experimental and control pits

In order to evaluate the influence of pit-entry fermentation on the quality of the resultant crude liquor, a sensory analysis was carried out by the experts well-trained in Chinese liquor tasting. It was obvious that the crude liquor obtained from the distillation of the experimental *Zaopei* displayed higher scores for acceptability (9.3), taste (8.7), smell (9.1), aftertaste (8.3) and acidity (7.9) as compared with the liquors distilled from the control pits (Fig. 8), which yielded lower sensory attribute scores. This phenomenon may be attributable to lower concentrations of ethyl lactate and higher concentrations of the other three esters—in particular the higher ethyl caproate levels present in the raw liquor. Higher concentrations of organic acids, such as caproic acid, acetic acid, and butyric acid, present in the experimental pit *Zaopei* may have also contributed to the liquor flavour and taste, as organic acids have been reported to help to make the flavor and taste of liquor harmonious and to increase liquor softness. These results allow us to conclude that the co-culture of *C. kluyveri* H068 and Methanogen 166 is a promising means of producing caproic acid that is potentially useful for liquor brewing applications.

## Additional file

**Additional file 1.** The layout of the 1000 L caproic acid co-culture fermentation pilot plant.1, N_2_ storage 2 tank; 2, CO_2_ storage tank; 3, H_2_ storage tank; 4, 5, Gas filter; 6, 35 L Methanogen 166 seed preparation reactor; 7, 70 L *C. kluyveri* H588 seed preparation reactor; 8, Pump; 9, Mixing tank; 10, Pump; 11, Plate heat exchanger; 12, Ethanol tank; 13, 1000 L reactor; 14, Pump; 15, Product storage tank; 16, Pump.
