# Supplementary material for: Improvement of caproic acid production in a Clostridium kluyveri H068 and Methanogen 166 co-culture fermentation system
Source: AMB Express. 2018 Oct 25;8:175. doi: 10.1186/s13568-018-0705-1 (PMC6202304; doi:10.1186/s13568-018-0705-1)
Supplement: Supplementary file 1 — Additional file 1. The layout of the 1000 L caproic acid co-culture fermentation pilot plant.1, N2 storage 2 tank; 2, CO2 storage tank; 3, H2 storage tank; 4, 5, Gas filter; 6, 35 L Methanogen 166 seed preparation reactor; 7, 70 L C. kluyveri H588 seed preparation reactor; 8, Pump; 9, Mixing tank; 10, Pump; 11, Plate heat exchanger; 12, Ethanol tank; 13, 1000 L reactor; 14, Pump; 15, Product storage tank; 16, Pump. [file 13568_2018_705_MOESM1_ESM.doc]

**Supplementary file 1**

The layout of the 1000 L caproic acid co-culture fermentation pilot plant.1, N_2_ storage tank; 2, CO_2_ storage tank; 3, H_2_ storage tank; 4, 5, gas filter; 6, 35 L Methanogen 166 seed preparation reactor; 7, 70 L *C. kluyveri* H588 seed preparation reactor; 8, Pump; 9, Mixing tank; 10, Pump; 11, Plate heat exchanger; 12, Ethanol tank; 13, 1000 L reactor; 14, Pump; 15, Product storage tank; 16, Pump.
